# Population Dynamics of *Mesocriconema xenoplax* Parasitizing Sweet Cherry Trees in British Columbia, Canada

**DOI:** 10.2478/jofnem-2024-0041

**Published:** 2024-11-23

**Authors:** Thomas Forge, Paige Munro

**Affiliations:** Agriculture and Agri-Food Canada, Summerland Research and Development Centre, Summerland, British Columbia

**Keywords:** climate change, host-parasitic relationship, nematode population dynamics, sampling, soil microclimate

## Abstract

The ring nematode, *Mesocriconema xenoplax*, has become recognized as a widespread pest of sweet cherry trees in the Okanagan Valley of British Columbia (BC). Understanding the cumulative impacts of *M. xenoplax* on tree health, interpreting diagnostic sample data, and predicting the impacts of climate change on *M. xenoplax* population densities all depend on knowledge of the temporal dynamics of *M. xenoplax* populations and their linkage with soil temperature and moisture regimes. The objective of this study was to measure population densities of *M. xenoplax* on a monthly basis over five years, in relation to soil temperature and moisture regimes, in a 16-year-old irrigated sweet cherry orchard. We tested the following hypotheses: (i) population densities would start low each spring and increase continuously with soil degree-day heat accumulation during each growing season, and (ii) year-to-year variation in population growth during the growing season would be correlated with year-to-year variation in soil degree-day heat accumulation. The data did not support these hypotheses and indicated that although there were significant differences in population densities among sample dates, there were no regular seasonal cycles of population growth and decline. We suggest that in mature cherry orchards, density-dependent processes mask the influences of annual changes in soil temperature and moisture on population processes. The data indicate that for diagnostic sampling purposes, all seasons would be equally representative of *M. xenoplax* population densities in irrigated orchards in BC. Furthermore, the lack of any strong linkage between soil temperature regimes and within- or across-year population dynamics indicate that modeling efforts based solely on abiotic drivers of temperature and moisture would not likely represent changes in population dynamics of *M. xenoplax* that will actually occur with climate change.

The ring nematode, *Mesocriconema xenoplax*, has recently been documented to be widespread in sweet cherry (*Prunus avium*) orchards in the Okanagan Valley of British Columbia (BC), which is at the northern limit of cherry production in North America (Forge et al., 2021). However, little is known of *M. xenoplax*’s effects on sweet cherry trees in more northern, cool-temperate environments, such as the Okanagan Valley. Knowledge of the temporal dynamics of parasitic nematode populations is needed to assess nematode impacts on crop health and to identify appropriate sampling times for diagnostic and monitoring purposes (Ferris et al., 2004). Understanding how nematode populations respond to variation in the soil microclimate is also crucial for anticipating longer-term changes in pest pressure resulting from climate change.

Previous studies of the population dynamics of *M. xenoplax* have been limited to warm-temperate regions, and few have sampled over more than one growing season. *M. xenoplax* is a damaging pest of other *Prunus* species fruit trees — such as peach (*P. persica*) and plum (*P. domestica*) — grown in lower-latitude, warm-temperate regions, such as California (Ferris et al., 2004; McKenry, 2022) and the southeastern United States (Nyczepir, 1991; Khanal et al., 2022). In central California, the buildup of *M. xenoplax* populations under young peach and almond trees replanted into fumigated soil was monitored over six years (Ferris et al., 2004). After a lag period during the first growing season, overall population densities increased through the second, third, and fourth years and then appeared to stabilize at around 5,000 to 10,000 *M. xenoplax*/L soil. Over one year of sampling in three vineyards in Spain, peak *M. xenoplax* population densities were observed to correspond with precipitation events in summer and fall (Pinochet and Cisneros, 1986). In a South Carolina peach orchard, an unspecified population of ring nematodes appeared to decline from April to May, plateaued through July, and then declined again from July to August of one year (Khanal et al., 2022).

In more northern, cool-temperate regions such as the Okanagan Valley, soil temperatures can go through annual fluctuations of 20°C or more, and depending on timing and extent of snow cover, the soil may freeze to 30-cm depth during winter. A common assumption is that plant-parasitic nematode populations in such environments undergo distinct seasonal cycles, but there are no data to substantiate this for *M. xenoplax* in orchards or vineyards. The presence of seasonal cycles was unclear from the extensive study of *M. xenoplax* in central California. The populations in most treatment combinations increased continuously through the second, third, and fourth years, and sampling intervals were too infrequent thereafter (Ferris et al., 2004).

Perennial fruit production in the Okanagan Valley is dependent on regular irrigation that maintains relatively constant orchard soil moisture contents through the growing season. Therefore, we hypothesized that the soil temperature regime would be the primary driver of annual *M. xenoplax* population dynamics. More specifically, we hypothesized that each spring, population densities would start low and increase with soil degree-day heat accumulation during each growing season. Year-to-year variation in population growth during the growing season would be correlated with year-to-year variation in soil degree-day heat accumulation. In order to test these hypotheses — and improve general knowledge of *M. xenoplax* population dynamics in BC cherry orchards — we measured *M. xenoplax* population densities every month for five years in relation to soil temperature and moisture regimes in an irrigated sweet cherry orchard.

## Materials and Methods

### Study site

The cherry orchard was located on the grounds of the Agriculture and Agri-Food Canada, Summerland Research and Development Centre (49°33′59.01″N; 119°38°56.24″W). The soil was classified as Osoyoos loamy sand, an Orthic Brown soil formed on glaciofluvial deposits (Wittneben, 1986). These soils, which are commonly planted to orchards in the region, have low organic matter contents (1.4%) and water-holding capacities. Water release curves — which relate volumetric moisture content to physiologically relevant water potential (measured in kPa) — were developed for this soil in a nearby orchard block and indicated volumetric moisture contents of 34.4% at saturation (−0.001 kPa), 19.5% at −10 kPa, 12.5% at −33 kPa, 9.1% at −100 kPa, and 5.4% at “permanent” wilting point (−1500 kPa) (Neilsen et al., 2016).

Prior to this study, preliminary analyses of nematodes at the site indicated the presence of a population of *Mesocriconema* that was morphologically consistent with *M. xenoplax*. Identification was confirmed via barcode sequence analysis of the D3 expansion region of 26S rDNA using the D2A-D3B primer pair (Al-Banna et al., 1997), and the sequence from one representative specimen was filed with GenBank (accession number MT602637.1). *Pratylenchus* spp. nematodes were also present at the site, but at relatively low population densities (< 10 *Pratylenchus*/100 cm^3^ soil).

### Orchard properties and maintenance

The block was planted in 2002 with “Staccato” and “Sentennial” varieties grafted onto Mazzard rootstock, in a 5- (between-tree) × 6-m (between-row) spacing. This combination of variety, rootstock, and spacing is typical for modern sweet cherry orchards in the region. An approximately 2-m wide “herbicide strip” under the trees was kept free of competing vegetation via annual spring application of indaziflam preemergence herbicide (Alion®, Bayer Crop Science Canada) and mid-summer application of glyphosate (Roundup®, Bayer Crop Science Canada), as is standard for cherry orchard production in the region. The trees were irrigated with understory micro-sprinklers on a weekly basis between May and mid-September of each year to maintain a relatively constant soil moisture regime. Pruning, fertility, and pest management practices were carried out according to standard cherry orchard management practices developed for the region (BC Tree Fruit Production Guide, https://www.bctfpg.ca/).

### Sampling design

**Figure 1:**
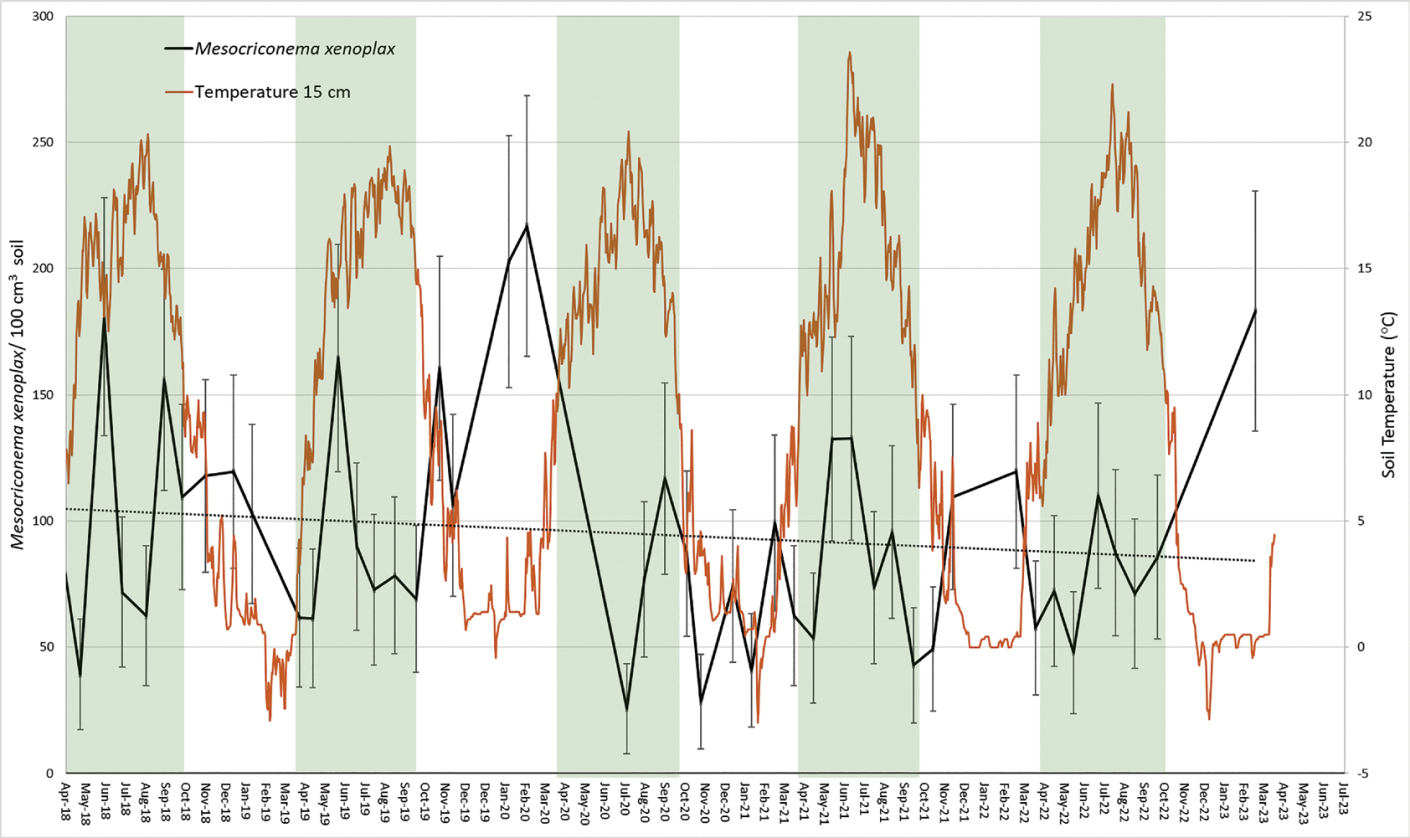
Temporal dynamics of *Mesocriconema xenoplax* population densities (*Mesocriconema xenoplax*/100 cm^3^ soil) in relation to soil temperature at 15-cm depth, through five consecutive population years under a 16- to 21-year-old cherry orchard. Shaded areas encompass the growing season, April to October. Data points are least-squared means and error bars are +/− standard errors from the overall repeated measures analysis of effect of sample date. The dotted line is a regression line for the entire data set (*R^2^* = 0.0014).

**Figure 2:**
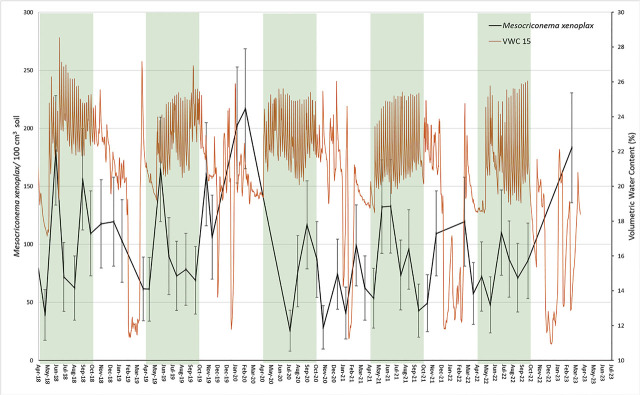
Temporal dynamics of *Mesocriconema xenoplax* population densities (*Mesocriconema xenoplax*/100 cm^3^ soil) in relation to volumetric soil moisture contents at 15-cm depth (VWC 15), through five consecutive population years under a 16- to 21-year-old cherry orchard. Shaded areas encompass the growing season, April to October. Data points are least-squared means and error bars are +/− standard errors from the overall repeated measures analysis of effect of sample date.

Given the relatively tight in-row spacing of modern sweet cherry orchards, we defined our sampling units to be row-plots rather than individual trees. Five adjacent rows, each 100 m long and comprising 20 trees, were designated to be the five row-plots for continuous sampling. A Decagon EM50 datalogger (METER Group, Inc., Pullman, WA) was installed in the middle of the third row-plot, and 5TE and 5TM probes (METER Group, Inc., Pullman, WA) were installed at 15-cm depth to record soil moistures and temperatures at hourly intervals. The 15-cm depth was the midpoint of nematode sample depth. Daily averages were computed and presented in Figures 1 and 2. Daily average temperatures at 15 cm were also used to calculate base 0°C degree-days, as a running sum of daily average temperatures above 0°C, for each year.

### Nematode sampling and extraction

Commencing 3 April 2018, a composite soil sample was taken from each of the five row-plots during the first week of each month through March 2023, completing five full years of sampling. Exceptions to the sampling frequency were April, May, and June 2020 when the COVID-19 pandemic prevented access to the research plots; and nine winter sample dates when soil was frozen (February and December 2019; January and December 2020; January, February, and December 2022; January and February 2023). Each sample representing a row-plot was comprised of five 2-cm-diam. × 30-cm deep cores taken using a JMC standard sample probe (Clements Associates, Inc., Newton, IA). One core was taken from each of five trees randomly picked each date from the 20 trees in each row-plot. We did not take multiple cores from around the base of particular trees because our sampling units were the row-plots, not individual trees, and we were trying to avoid damage to root systems that could occur from such intensive sampling over multiple years. To ensure that each composite sample equally represented the tree root zone within the herbicide strip, all five cores were taken 50 cm out from tree trunks: two in line with the tree row, another two at a 45-degree angle out from the tree row axis, and one 90 degrees out from the axis of the tree row. The 30-cm sample depth was chosen to encompass the 10- to 20-cm soil depth interval in which maximum fine cherry roots densities have been measured (Li et al., 2019). Additionally, the 0- to 30-cm depth interval contained 70% of the total *M. xenoplax* population in the soil profile in a Washington vineyard (Howland et al., 2014). A different set of five trees within each row-plot was sampled at each date to minimize the number of cores taken from any individual tree over the course of the study.

Nematodes were extracted from 100 cm^3^ aliquots of each sample using a modified wet sieving-sucrose flotation method (Jenkins, 1964), and *Mesocriconema* and *Pratylenchus* nematodes were counted using a Meiji Techno TC5100 inverted microscope (Meiji Techno America, Campbell, CA) at x40 magnification.

### Data analyses

**Figure 3:**
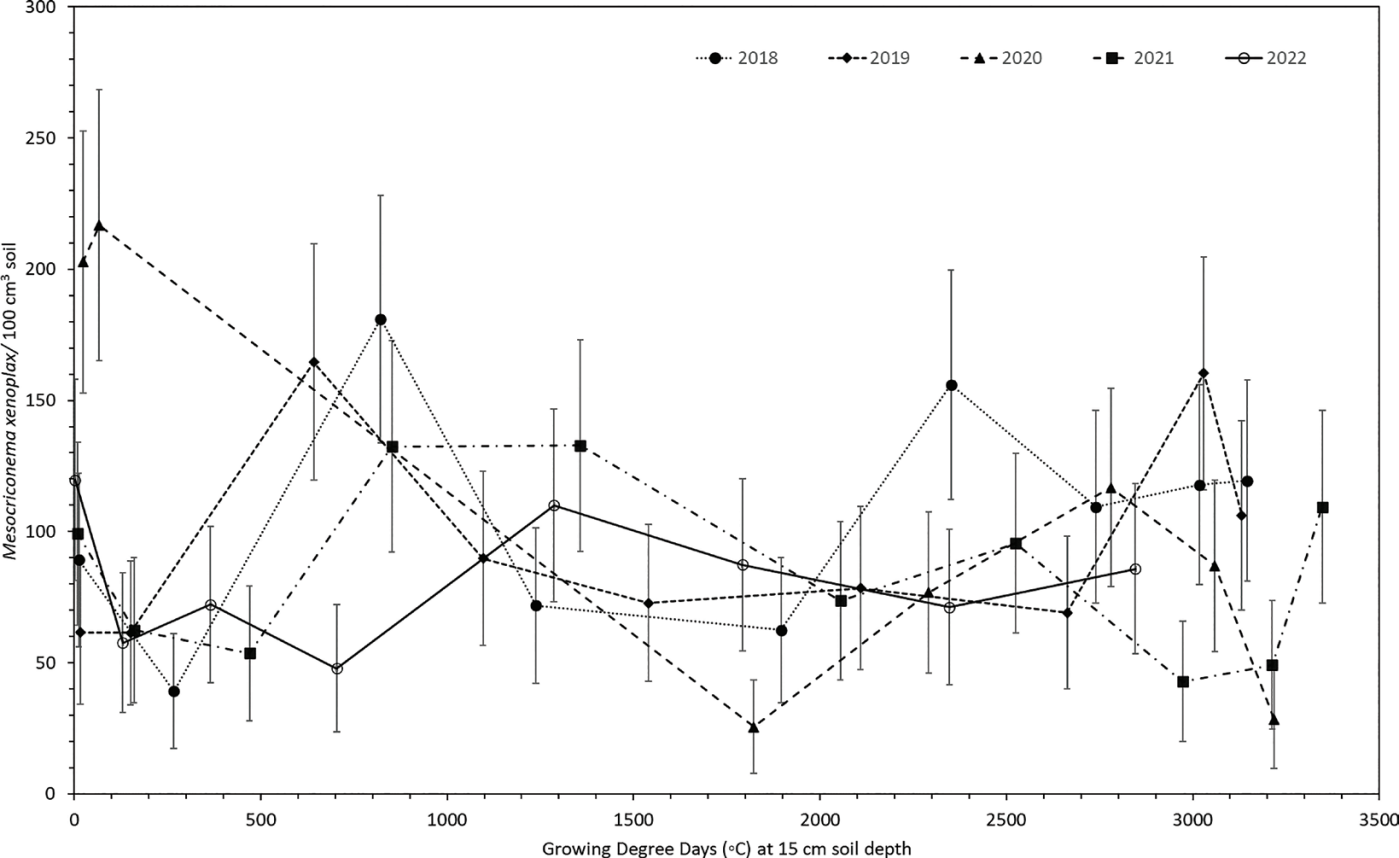
Population densities of *Mesocriconema xenoplax* (*Mesocriconema xenoplax*/100 cm^3^ soil) in relation to accumulation of soil degree-days at 15-cm depth calculated using base 0°C, for each of five growing seasons in a 16- to 21-year-old sweet cherry orchard in British Columbia.

For purposes of data analyses, we defined population years as April through March and growing seasons as April through October. The raw *M. xenoplax* count data were first subjected to tests of normality using Proc Univariate in SAS (SAS Institute, Inc., Cary, NC), and were found to not be normally distributed. Data were subsequently subjected to a generalized linear mixed model analysis, using Proc GLIMMIX in SAS, with sample dates coded as repeated measures within each row-plot and the row-plot coded as a random effect variable. The model assumed Poisson distribution and used the log-link function to accommodate the nonnormal nematode count data. Back-transformed, least-squared means and standard errors were computed for sample dates. These analyses were conducted on the entire data set, for each population year, and for each growing season within each year. Least-squared means and standard errors for each sample date were plotted in Figures 1, 2, and 3. Simple linear regression in Excel was used to test for an overall trend in population densities across the five years.

## Results

Soil temperatures at 15-cm depth rose above 10°C in late April of most years and then dropped to below 10°C in mid-October of most years (Table 1; Fig. 1). The 2021 growing season was the hottest, with the maximum soil temperature at 15-cm depth of 23°C reached on July 3, nearly a month earlier than other yearly maxima; it was also overall the warmest, with accumulation of 3379 degree-days at 15-cm depth (Table 1).

**Table 1:** Summary of soil temperatures at 15-cm depth and seasonal milestones for the five population years (April 1 through March 31) of the study. Column headings “Date > 10°C” and “Date < 10°C” are the dates on which soil temperatures rose above 10°C or dropped below 10°C, respectively. “Max. (date)” and “Min. (date)” refer to maximum and minimum temperatures recorded within each population year and the dates they were recorded. “DD total” refers to the total number of degree-days accumulated through the year, calculated using base 0°C.

**Year**	**Date > 10°C**	**Max. (date)**	**Date < 10°C**	**DD total**	**Min. (date)**
2018–19	21 April	20 (11 Aug)	3 Oct	3172	−2.9 (10 Feb)
2019–20	18 April	20 (10 Aug)	8 Oct	3234	−0.4 (15 Jan)
2020–21	18 April	20 (1 Aug)	13 Oct	3305	−3.0 (12 Feb)
2021–22	16 April	23 (1 July)	8 Oct	3379	0 (27 Dec)
2022–23	29 April	22 (28 July)	18 Oct	3190	−2.9 (22 Dec)

Regularly scheduled irrigation maintained volumetric soil moisture contents between 20% and 26% during each growing season (Fig. 2). These values are well above the estimated 12.5% water contents measured for this soil at field capacity (−33kPa suction), but below saturation content of 34%, reflecting the management objective of maintaining soil moisture in the range optimal for tree growth. Distinct declines in moisture contents occurred each winter as surface layers of the soil froze, causing freezing-induced redistribution of soil moisture (Fig. 2).

Over the 47 sample dates spanning April 2018 through March 2023, *M. xenoplax* population densities ranged from a minimum of 26 *M. xenoplax*/100 cm^3^ soil in July 2020 to a maximum of 217 *M. xenoplax*/100 cm^3^ soil in March 2020 (Table 2; Figs. 1,2). The repeated measures analysis indicated significant changes in *M. xenoplax* population densities over the five years (sample date effect *p* = 0.001; Fig. 1). However, when analyses were broken down by year, the date effect was significant in 2020 only (*p* = 0.04; Table 2), and when broken down by growing season within each year, the date effect was significant during the growing season of 2018 only (*p* = 0.04; Table 2). Regression analysis did not indicate any significant overall change across the five years (*R^2^* = 0.001; Fig. 1).

**Table 2:** Maximum, minimum, and overall mean population densities of *Mesocriconema xenoplax* (*M. xenoplax*/100 cm^3^ soil) for each of the five population years (April 1 through March 31, variable number of sample dates per year) in a sweet cherry orchard in Summerland, British Columbia. Month names in parentheses are the month in which the maximum or minimum was recorded. The two “Date *p*-values” for each year are p-values from repeated measures analyses of effect of sample date for the entire population year and for the growing season (April through October), respectively.

**Year**	**Maximum**	**Minimum**	**Mean**	**Date *p*-value**
2018–19 (*n* = 55)	181 (June)	39 (May)	101	0.08, 0.04
2019–20 (*n* = 50)	217 (March)	61 (April)	122	0.06, 0.09
2020–21 (*n* = 40)	117 (September)	26 (July)	69	0.04, 0.15
2021–22 (*n* = 50)	133 (July)	43 (October)	87	0.13, 0.13
2022–23 (*n* = 40)	183 (March)	48 (June)	89	0.20, 0.18

In terms of trends within years, *M. xenoplax* population densities did not consistently start from a low point each spring and increase with accumulation of soil degree-days during the growing season (Fig. 3). In the five population years (April through March), maximum population densities were observed in June, March, September, July, and March of the 2018–19, 2019–20, 2020–21, 2021–22, and 2022–23 population years, respectively (Table 2). Similarly, minimum population densities were observed in May, April, July, October, and June of the 2018–19, 2019–20, 2020–21, 2021–22, and 2022–23 population years, respectively (Table 2). Accordingly, there was also no clear relationship between winter minimum soil temperatures or freezing events and the occurrence of springtime minimum population densities (Fig. 1).

Average annual *Pratylenchus* population densities for the five years of the study were 10, 10, 2, 5, and 12 *Pratylenchus*/100 cm^3^. Because they remained consistently low, they were not analyzed further.

## Discussion

Contrary to our working hypothesis, we found that population growth of *M. xenoplax* did not start low each spring, as a result of overwinter mortality, and did not increase continuously during each growing season in relation to soil degree-day accumulation. Among the five population years, minimum annual population densities were observed in all seasons except winter, and maxima were observed in all four seasons. We have ruled out soil moisture fluctuations as a primary driver of changes in population densities in this system; regular irrigation maintained growing season soil moisture contents within an optimal range for tree growth, which we presume would be optimal for nematode activity. The only distinct declines in soil moisture were associated with winter freezing of surface layers. As for minimum soil temperatures, these seasonal declines in soil moisture were not linked with the occurrence of subsequent *M. xenoplax* population minima or maxima. In addition to the lack of a predictable seasonal trend, there was no overall trend in population densities over the five-year period.

The lack of a predictable seasonal trend in population dynamics suggests that the *M. xenoplax* population may have reached a dynamic equilibrium with root systems of the host trees, which were 16 years old when the study began. Under such conditions, the interplay of density-dependent factors — such as fluctuating availability of fine roots or the abundance of nematode predators or parasites (Jaffee et al., 1989; Ferris et al., 2004) — could influence population dynamics, masking direct effects of soil temperatures on population growth. The lack of a distinct seasonal trend in population dynamics under a temperate perennial crop has been noted in the case of *Pratylenchus penetrans* parasitizing raspberry in the Fraser Valley of BC (Vrain et al., 1996; Forge et al., 1998). Fluctuations in *P. penetrans* population densities over two years did not follow seasonal patterns despite seasonal variation in soil temperatures of −2.5°C to 23°C (Vrain et al., 1996; Forge et al., 1998), but the authors were unable to detect a relationship between fluctuations in fine root densities and *P. penetrans* population dynamics. Ferris et al. (2004) measured frequency of parasitism of *M. xenoplax* by the nematode-parasitic fungus *Hirsutella rhossiliensis*, but the authors did not observe sufficient levels of parasitism to suggest regulation of the *M. xenoplax* population (perhaps because it was still building up on the relatively young plum trees and had not yet fully reached dynamic equilibrium with either host roots or the *H. rhossiliensis*). Neither fluctuations in fine root densities nor abundances of nematode-parasitizing fungi or predacious nematodes or mites were monitored in the present study.

We suggest that, in addition to density-dependent factors, random sampling-related variation could underly much of the seemingly “significant” variation in population densities among individual sample dates. For example, the five cores taken from each 100-m row of trees at each date may have been inadequate to accurately represent a population that varied spatially within the tree-row. Such inadequate sampling could have contributed to the apparently random occurrence of date-to-date variation in population densities. More detailed knowledge of spatial distributions of *M. xenoplax* in orchards — and sample-to-sample variation at different spatial scales and sampling intensities (e.g., number of cores per unit area per composite sample) relative to analytical sources of variation (Lawrence and Zehr, 1978) — is needed. More detailed knowledge of the vertical distribution of *M. xenoplax* is also needed. A previous study indicated that the majority of *M. xenoplax* are located in the top 30 cm of vineyard soil (Howland et al., 2014) at one date, but seasonal changes in vertical distribution have not been studied. Such knowledge would provide a more solid basis for interpreting variation among samples taken for general monitoring purposes as well as for field experiments, and it would facilitate development of improved sampling and sample-processing protocols.

Controlled greenhouse and laboratory experiments have shown that development and reproduction of southern populations of *M. xenoplax* increases with temperatures above 10°C (Lownsbery, 1959, 1961; Seshadri, 1965; Westcott and Burrows, 1991). No prior controlled experiments have assessed development or reproduction at temperatures below 10°C, which is approximately half of each calendar year in BC. While there was no discernable annual cycle in overall *M. xenoplax* population densities, we suggest that the life cycle may nonetheless follow an annual cycle. Future analyses should assess the life-stage distribution of the population at each sample date to determine when egg hatch occurs and to identify the number of generations each year. Such data could provide additional insight to the possible influences of temperature, and by extension climate change, on *M. xenoplax* population dynamics in cool-temperate environments.

As the effects of climate change on agroecosystems become increasingly evident, there is growing interest in predicting changes in population dynamics and impacts of pests. Based on data from laboratory, growth chamber, and greenhouse studies with annual crop plants, climate change has been predicted to increase population growth or virulence of plant-parasitic nematodes (Khanal et al., 2023). Our field-based study did not reveal any strong linkage between soil temperature regimes and within- or across-year population dynamics of *M. xenoplax*. This result suggests that modeling efforts based primarily on the abiotic drivers of temperature and moisture are not likely to accurately represent the changes in population dynamics of *M. xenoplax* that will actually occur with climate change.

The notion of a pest population being in dynamic equilibrium with its host implies a characteristic carrying capacity of the host. It is worth noting that the California study of *M. xenoplax* population dynamics (Ferris et al., 2004) indicated similar equilibrium population densities of 5,000 to 10,000 *M. xenoplax*/L soil for the “tolerant” Lovell and the “susceptible” Nemaguard peach rootstocks, although the rate of initial population development appeared to be greater for Nemaguard rootstock. In the present study, which employed similar extraction procedures as the California study, the average *M. xenoplax* population density over the 47 sample dates was 951 *M. xenoplax*/L soil. These substantial differences in apparent carrying capacity between California peach and BC cherry orchards suggest differences in inherent host status or carrying capacity of the peach (Lovell and Nemaguard) and cherry (Mazzard) rootstocks. However, geographical differences in soil physicochemical properties and soil ecology (e.g. antagonists) could also underly such substantial differences in apparent carrying capacity between the two sites. The effect of *M. xenoplax* on sweet cherry tree health in BC has not yet been demonstrated experimentally. Future experiments to compare population development of *M. xenoplax* on a range of cherry and peach rootstocks under identical environmental conditions would help determine the reason for such substantial differences in apparent carrying capacity and provide insight into the potential impacts of *M. xenoplax* on cherry orchards in BC and similar northern-temperate production areas.
